# MiR-106b-5p regulates esophageal squamous cell carcinoma progression by binding to HPGD

**DOI:** 10.1186/s12885-022-09404-8

**Published:** 2022-03-22

**Authors:** Fan Yang, Zhanwen Sun, Dengyun Wang, Tian Du

**Affiliations:** Department of Thoracic and Cardiovascular Surgery, Huangshi Central Hospital, Affiliated Hospital of Hubei Polytechnic University, Edong Healthcare Group, No. 114, Tianjin Street, Huangshi, 435000 Hubei P.R. China

**Keywords:** MiR-106b-5p, 15-hydroxyprostaglandin dehydrogenase, Esophageal squamous cell carcinoma, Proliferation, Colony formation, Adhesion, Migration, Invasion, Cell cycle, Apoptosis

## Abstract

**Background:**

Several studies have documented the key role of microRNAs (miRNAs) in esophageal squamous cell carcinoma (ESCC). Although the expression of the 15-hydroxyprostaglandin dehydrogenase (*HPGD*) gene and miR-106b-5p are reportedly linked to cancer progression, their underlying mechanisms in ESCC remain unclear.

**Methods:**

mRNA and miRNA expression in ESCC tissues and cells were analyzed using RT-qPCR. Luciferase and RNA pull-down assays were used to identify the interaction between miR-106b-5p and HPGD. Xenograft and pulmonary metastasis models were used to assess tumor growth and metastasis. CCK-8, BrdU, colony formation, adhesion, cell wound healing, Transwell, and caspase-3/7 activity assays, and flow cytometry and western blot analyses were used to examine the function of miR-106-5p and HPGD in ESCC cell lines.

**Results:**

The findings revealed that miR-106b-5p expression was upregulated in ESCC tissues and cell lines. miR-106b-5p augmented cellular proliferation, colony formation, adhesion, migration, invasion, and proportion of cells in the S-phase, but reduced apoptosis and the proportion of cells in G1-phase. Silencing of miR-106-5p inhibited tumor growth in vivo and pulmonary metastasis. Although HPGD overexpression suppressed proliferation, colony formation, adhesion, migration, and invasion of ESCC cells, it promoted apoptosis and caused cell cycle arrest of the ESCC cells. The results also indicated a direct interaction of HPGD with miR-106b-5p in ESCC cells. Furthermore, miR-106b-5p inhibited HPGD expression, thereby suppressing ESCC tumorigenesis.

**Conclusion:**

Our data suggest that miR-106b-5p enhances proliferation, colony formation, adhesion, migration, and invasion, and induces the cycle progression, but represses apoptosis of ESCC cells by targeting HPGD. This suggests that the miR-106b-5p/HPGD axis may serve as a promising target for the diagnosis and treatment of ESCC.

**Supplementary Information:**

The online version contains supplementary material available at 10.1186/s12885-022-09404-8.

## Background

Esophageal cancer (EC), a malignancy that affects the esophagus, has a five-year survival rate of less than 20% [1, 2] and has become a public health concern. Esophageal squamous cell carcinoma (ESCC) accounts for approximately 80% of ECs and constitutes the fastest-growing EC subtype in East Asia [3, 4]. Although the therapeutic strategies for ESCCs have improved, poor prognosis and treatment of patients with this cancer have become a major concern for stakeholders in the health sector [5]. In addition to chemotherapy and radiotherapy, traditional surgical techniques often fail to prevent the metastatic spread and recurrence of this cancer. Therefore, there is an urgent need to explore novel targets that may serve as effective therapeutic biomarkers for ESCC.

In this study, we sought to identify and characterize the molecules that contribute to the development of ESCC. First, we identified 15-hydroxyprostaglandin dehydrogenase (HPGD) gene located on chromosome 4q34.1 as a potential candidate. The *HPGD* gene consisting of 10 exons encodes an alcohol dehydrogenase protein, and participates in the metabolism of prostaglandins and in other cellular processes [6, 7]. The *tumor* suppressive role of HPGD has been observed in several cancers [8–10]. A previous study reported decreased expression of HPGD in ESCC tissues [11]. However, the relationship between HPGD and ESCC requires further investigation.

MicroRNAs (miRNAs) have also been linked to cancer progression. They are small single stranded non-coding RNA molecules (containing approximately 23 nucleotides) that perform their biological functions by binding to target mRNAs. As post-transcriptional regulators, miRNAs impair the stability of their target mRNAs, resulting in translational inhibition [12, 13]. Several miRNAs are associated with cancer processes, and have also been identified as potential diagnostic markers in various human cancers [14, 15]. In this study, using through bioinformatics analyses we screened two crucial miRNAs (miR-31-5p and miR-106b-5p) that may target HPGD and enable ESCC progression. According to the data from the starBase, miR-106b-5p expression is more robustly upregulated compared to that of miR-31-5p in ESCC samples, and therefore we focused on the role of miR-106b-5p in ESCC. In fact, miR-106b has been extensively studied since 2008, and many studies have shown the critical biological functions of miR-106b in tumorigenesis, such as in cell proliferation, metastasis, and apoptosis [16–18], anti-miR-106b has been proposed as a promising approach for cancer therapy [16, 19, 20]. Other cancers linked to miR-106b-5p include colorectal, breast, and gastric carcinomas [21–23]. Downregulation of miR-106b augments ESCC tumorigenesis by promoting cell proliferation and epithelial-mesenchymal transition (EMT) [24–26]. However, only one study has shown that miR-106b-5p also promotes cell migration and invasion by enhancing EMT in ESCC [25]. Other potential mechanisms underlying miR-106b-5p-mediated ESCC remain unclear.

This study aimed to investigate the role of the miR-106b-5p/HPGD axis in ESCC cell progression in vitro with the objective of providing insights for ESCC therapies.

## Materials and methods

### Microarray analysis

Two mRNA (GSE38129 and GSE17351) and one miRNA (GSE114110) expression profile was downloaded from the GEO DataSet (https://www.ncbi.nlm.nih.gov/gds/). GSE38129 included ESCC and adjacent normal samples from 30 Chinese patients, whereas GSE17351 included ESCC and adjacent normal samples from five American patients. With adjusted *P*-value (adj. P) < 0.05, and log fold change (logFC) < − 1, the differentially expressed genes (DEGs) were screened using the limma 3.26.8 package. For the miRNAs, Limma 3.26.8 was applied to analyze the differentially expressed miRNAs in ESCC with adj. *P* < 0.05, and logFC > 1. Metascape (https://metascape.org/gp/index.html) was used to analyze the key biological processes associated with these DEGs. Their expression in normal and esophageal carcinoma (ESCA) tissues was analyzed using UALCAN, with data obtained from the cancer genome atlas (TCGA). StarBase (http://starbase.sysu.edu.cn) was used to analyze miRNA expression in ESCA tissues and to predict the miRNAs that bind to HPGD. TargetScan was also used to predict the miRNAs that could bind to HPGD. Finally, DEGs and miRNAs were overlapped using the Venny 2.1.0.

### Tissue samples and cell lines

ESCC tissue specimens (*n* = 45) and paired adjacent normal esophageal samples (*n* = 45) were collected from patients at Huangshi Central Hospital, Wuhan, China between October 2016 and February 2019. The protocol was approved by the Ethics Committee of the Huangshi Central Hospital, and all patients read and completed the informed consent forms to participate in the study. All tissues were stored at − 80 °C, and the clinical data pertaining to individual patients with ESCC are listed in Table 1.

**Table 1:** Clinical characteristics of 30 ESCC patients

Characteristics	*N* = 45	miR-106b-5p expression		*P*	HPGD expression		*P*
		Low (23)	High (22)		Low (23)	High (22)	
Gender				0.181			0.626
Male	27	16	11		13	14	
Female	18	7	11		10	8	
Age (years)				0.349			0.758
> 50	24	15	9		11	13	
≤ 50	21	8	13		12	9	
Histology differentiation				0.018			0.013
Poorly	14	3	11		8	6	
Middle	22	13	9		7	15	
Well	9	7	2		8	1	
Weight loss				0.302			0.167
> 5%	19	8	11		12	7	
≤ 5%	26	15	11		11	15	
TNM stage				0.014			0.013
Stage I	15	12	3		3	12	
Stage II	21	9	12		14	7	
Stage III	9	2	7		6	3	
KPS				0.048			0.017
> 90	29	18	11		11	18	
70–90	16	5	11		12	4	

*KPS* Karnofsky Performance Status

Human ESCC cell lines (KYSE30, KYSE180, KYSE450, and KYSE510) and normal esophageal epithelial cells (Het-1A) were purchased from ATCC (Manassas, VA, USA). All cell lines used in this study were cultured in Dulbecco’s modified Eagle’s medium (DMEM; Cat#: A4192101, Gibco, Waltham, MA, USA) containing 10% fetal bovine serum (Cat#: 10099141, Gibco) and incubated at 37 °C in an incubator containing 5% CO_2_.

### RNA isolation and RT-qPCR

RT-qPCR and RNA isolation were performed according to methods described previously [27]. RNA isolation from the cell samples was performed using TRIzol Reagent (Invitrogen, Waltham, MA, USA). cDNA was obtained from the extracted RNA using the PrimeScript First Strand cDNA Synthesis Kit (Takara, Dalian, China). Gene expression was quantitated by RT-qPCR using SYBR Premix Ex Taq (Takara) on an ABI Prism 7900 Detector System (Life Technologies Inc., Waltham, MA, USA). miRNA and mRNA expressions were normalized to that of U6 and β-actin, respectively. The data were analyzed using the 2^−ΔΔCT^ method. The primer sequences used are listed in Table 2.

**Table 2:** The primer sequences for RT-qPCR

GENE	Primer sequences (5′-3′)
miR-106b-5p	Forward: TGCGGCAACACCAGTCGATGG
	Reverse: CCAGTGCAGGGTCCGAGGT
U6	Forward: *ATTGGAACGATACAGAGAAGATT*
	Reverse: *GGA ACGCTTCACGAATTTG*
HPGD	Forward: CTCTGTTCATCCAGTGCGAT
	Reverse: CTCCCGAGTAAAGGACCCACA
MAL	Forward: TCTTTTACCTCAGCGCCTCA
	Reverse: CGGCCAGTTAACACCATCTG
GAPDH	Forward: AGCCACATCGCTCAGACAC
	Reverse: GCCCAATACGACCAAATCC

### Cell transfection

MiR-106b-5p inhibitor, miR-106b-5p mimic, and their corresponding negative controls, were purchased from Shanghai Tuoran Co., Ltd. The corresponding sequences are listed in the Supplemental Table 1. A full-length HPGD cDNA was synthesized (Shanghai Tuoran Co. Ltd.) and cloned into pcDNA3.1 plasmid. Puromycin (4 μg/mL) was used to select stably transfected cells. KYSE450 and KYSE510 cells (3 × 10^5^ cells) were transfected with 50 nM miR-106b-5p inhibitor, miR-106b-5p mimic, or miR-NC using Lipofectamine 3000 Reagent (Invitrogen, Waltham, MA, USA). The cells were cultured for 48 h before performing subsequent experiments as described previously [28].

### Cell counting Kit-8 (CCK-8) assay

CCK-8 assay was performed to investigate cell viability [29] (Cat#: K1018; APExBIO, China). Briefly, KYSE450 and KYSE510 cells (3 × 10^3^ cells) were seeded in a 96-well plate. At the indicated times, CCK-8 (10 μL) reagent was added to the wells with cells, and the cells were incubated further for 2 h. Finally, the optical density (OD) was measured at 450 nm with a multimode-plate-reader (Tecan, Switzerland).

### BrdU assay

BrdU assay was performed according to a previously reported method [30]. First, KYSE450 and KYSE510 cells (3 × 10^3^ cells) were cultured in 96-well plates for 24 h followed by 12 h of serum starvation. After another 8 h, serum was added back to the cells. The BrdU Cell Proliferation Assay Kit (Cat#: 6813, CST, Danvers, MA, USA) was used to label cells for 8–12 h without removing the treatment media. Finally, the OD was measured at 450 nm.

### Cell adhesion assay

Cell adhesion assays were performed based on the methodology used in a previous study [22]. KYSE450 and KYSE510 cells (3 × 10^3^ cells) were cultured in 96-well plates. Collagen I solution (40 μg/mL; Cat#: C7661, Sigma-Aldrich, St. Louis, MO, USA) was added to the wells and the plates were stored overnight at 4 °C. The transfected cells were cultured in serum-free DMEM for 8 h. Cells were treated with 10 mM EDTA (in DMEM) for 10 min to dissociate them from the dishes. After collecting and resuspending the cells in DMEM with 0.1% BSA (2 × 10^5^ cells/mL), the cells suspension (100 μL) was added to a air-dried 96-well plate for another 30 or 60 min. After incubation, 100 μL DMEM was added to remove the non-adherent cells and the dishes were further incubated for 4 h. Subsequently, the MTT substrate (Cat#: CT01, Sigma) (10 μL/well) was applied to the treated cells for 2 h at 30 °C. Next, 100 μL of DMSO was added to each well containing the lysed cells. Finally, the absorbance was measured at 570 nm.

### Colony formation assay

Cells were disassociated, suspended, and plated in a 6-well culture plate at a density of 100 cells/well. After culturing for 14 days at 37 °C, the cells were washed twice with PBS and stained with Giemsa solution. Colonies larger than 75 μm in diameter or containing more than 50 cells were counted as a positive colony.

### Wound healing assay

Cells were plated in a 6-well plate and once they reached more than 90% confluence, the cell monolayer was scratched with a 200 μL pipette tip to produce a wound. After removing the floating cells, fresh medium (without serum) was added to the wells and the cells were cultured at 37 °C for 24 h. Images of cells at the same location were captured at 0 h and 24 h using an Olympus IX51 inverted microscope (Olympus, Tokyo, Japan), and the wound healing rate was calculated as percent wound width covered = (width at 0 h - width at 24 h)/width at 0 h × 100%.

### Cell invasion assay

Transwell inserts coated with Matrigel (40 μg/well, BD Biosciences, San Jose, CA, USA) were used to assess cell invasion. Cells (2 × 10^5^) in serum-free medium were added to the upper chamber of the Transwell, and 600 μL of medium containing 10% FBS was added to the lower chamber. After 48 h of culture at 37 °C, the cells that had penetrated the membrane and adhered to the surface of the lower membrane were fixed with methanol, stained with Giemsa, and photographed under a light microscope (Leica Microsystems, Wetzlar, Germany).

### Cell cycle analysis

Cells were collected 48 h after transfection and fixed overnight in 70% ethanol at 4 °C. Next, the DNA was stained using a cell cycle detection kit (KeyGen, Nanjing, China). Briefly, the cells were treated with RNase A and stained with 50 μg/mL propidium iodide. The DNA content was analyzed by flow cytometry using a FACS Calibur system (Becton Dickinson, Franklin, NJ, USA). Data were collected and processed using FlowJo FACS analysis software (Tree Star, Ashland, OR, USA).

### Caspase-3/7 activity assay

The apoptotic ability of ESCC cells was assessed using the caspase-3/7 Assay Kit (Cat#: G8090, Promega, Madison, Wisconsin, USA) as described previously [27]. According to the manufacturer’s guidelines, both the ESCC cell lines (3 × 10^3^ cells) were cultured in 96-well plates. Next, the detection solution was prepared, and caspase 3/7 buffer was added to the bottle containing the caspase 3/7 substrate. After adding the mixed solution (100 μL/well) to the 96-well plate containing cells at 80% cell density, the mixture was incubated at room temperature for 2 h. Finally, OD values were measured at 450 nm.

### Animal studies

Five-week-old female nude mice (5-weeks old) purchased from Shanghai SIPPR-BK Laboratory Animal Co. Ltd. (Shanghai, China) for the in vivo studies. Animal experiments were carried out in strict accordance with the Regulations for the Administration of Affairs Concerning Experimental Animals. KYSE510 cells (2 × 10^6^) from the inhibitor-NC and miRNA inhibitor transfected groups were collected and resuspended in 2 mL PBS. Cells were injected subcutaneously into the backs of the nude mice. Tumor size was measured with calipers every fifth day. The tumor volume (V) was calculated using the following formula: V = length × Width^2^ × 1/2. All the mice were euthanized 25 days after implantation by asphyxiation with carbon dioxide. The mice were placed into the euthanasia chamber and filled with CO_2_ at 30% chamber volume /min. When the mice were unconscious and stopped breathing, the CO_2_ flow was maintained for 1 min. The mice death was confirmed as cardiac arrest and did not respond to the toe-pinching reflex [28]. Tumors from the resected mice were weighed and photographed immediately.

The lung tissues were resected, fixed in 10% formaldehyde solution, dehydrated in an ethanol gradient, embedded in paraffin, and cut into slices of 4 μm thickness. After deparaffinization, the samples were stained with haematoxylin and eosin. The slices were then mounted and observed under a light microscope (Leica Microsystems).

### Luciferase assay

The pmiRGLO-HPGD3′-UTR-Wt and pmiRGLO-HPGD3′-UTR-mutated vectors were constructed by Guangzhou Boxin Biotechnology Co. Ltd. (China). KYSE450 and KYSE510 cells (3 × 10^5^ cells) were co-transfected with pmiRGLO HPGD 3′-UTR-Wt or HPGD 3’UTR-Mut and either miR-NC or miR-106b-5p. At post 48 h post-transfection, luciferase was assessed with the Luciferase Assay Kit (Cat#: #16185, Thermo Scientific, Waltham, MA, USA) according to the manufacturer’s instructions and normalized to the firefly luciferase levels used as an internal control.

### RNA pull down assay

Biotin-labeled negative control (Bio-NC) and miR-106b-5p (Bio-miR-106b-5p) used in this study were provided by RiboBio (China). The two Bio-miRNA mimics were first transfected into KYSE450 and KYSE510 cells. After for 48 h lysis buffer supplemented with the protease and RNase inhibitors was added to the cells. Streptavidin beads (Cat#: #88817, Thermo Scientific) were washed and added to the cell lysates. The cells were incubated overnight at 4 °C. Subsequently, the beads were washed twice, and the RNA was eluted and purified using the RNeasy Mini Kit (Cat#: 74104, QIAGEN, Germany). Finally, HPGD enrichment was detected by RT-qPCR analysis.

### Western blotting analysis

Proteins from the cells were denatured and quantified. First, proteins (30 μg) were separated on a 10% sodium dodecyl sulfate polyacrylamide gel electrophoresis (SDS-PAGE) gel. Next, the gels were electroblotted onto polyvinylidene difluoride (PVDF) membranes for hybridization. After blocking with 5% milk, the membranes were incubated with the following antibodies: anti-HPGD (1:1000, Cat#: ab187160, Abcam, UK), anti-Bax (1:2000, Cat#: ab32503, Abcam), anti-Bcl-2 (1:2000, Cat#: ab182858, Abcam), and β-actin (1:2000, Cat#: ab8226, Abcam) overnight at 4 °C. Then, the membranes were incubated with the corresponding secondary antibodies; goat anti-rabbit IgG H&L (HRP) (1:10000, Cat#: ab97051, Abcam) for HPGD and goat anti-mouse IgG H&L (HRP) (1:10000, Cat#: ab175783, Abcam, UK) for β-actin, at room temperature for 3 h. Next, ECL reagents (Bio-Rad, California, USA) were used to detect the protein bands. Finally, the density in each band was quantified using *Image J* software (*ImageJ* 1.48v, NIH, Maryland, USA).

### Statistical analysis

Experimental data presented as the means ± standard deviation (SD) were analyzed using the paired Student’s *t*-tests for two-group comparisons and one-way ANOVA with Dunnett’s post hoc for multiple group comparisons using SPSS software (version 19.0; IBM Corp., Armonk, NY, USA). Three independent repeats were performed for each experiment. Significance was set at *p* < 0.05.

## Results

### Involvement of HPGD and miR-106b-5p in ESCC

**Fig. 1:**
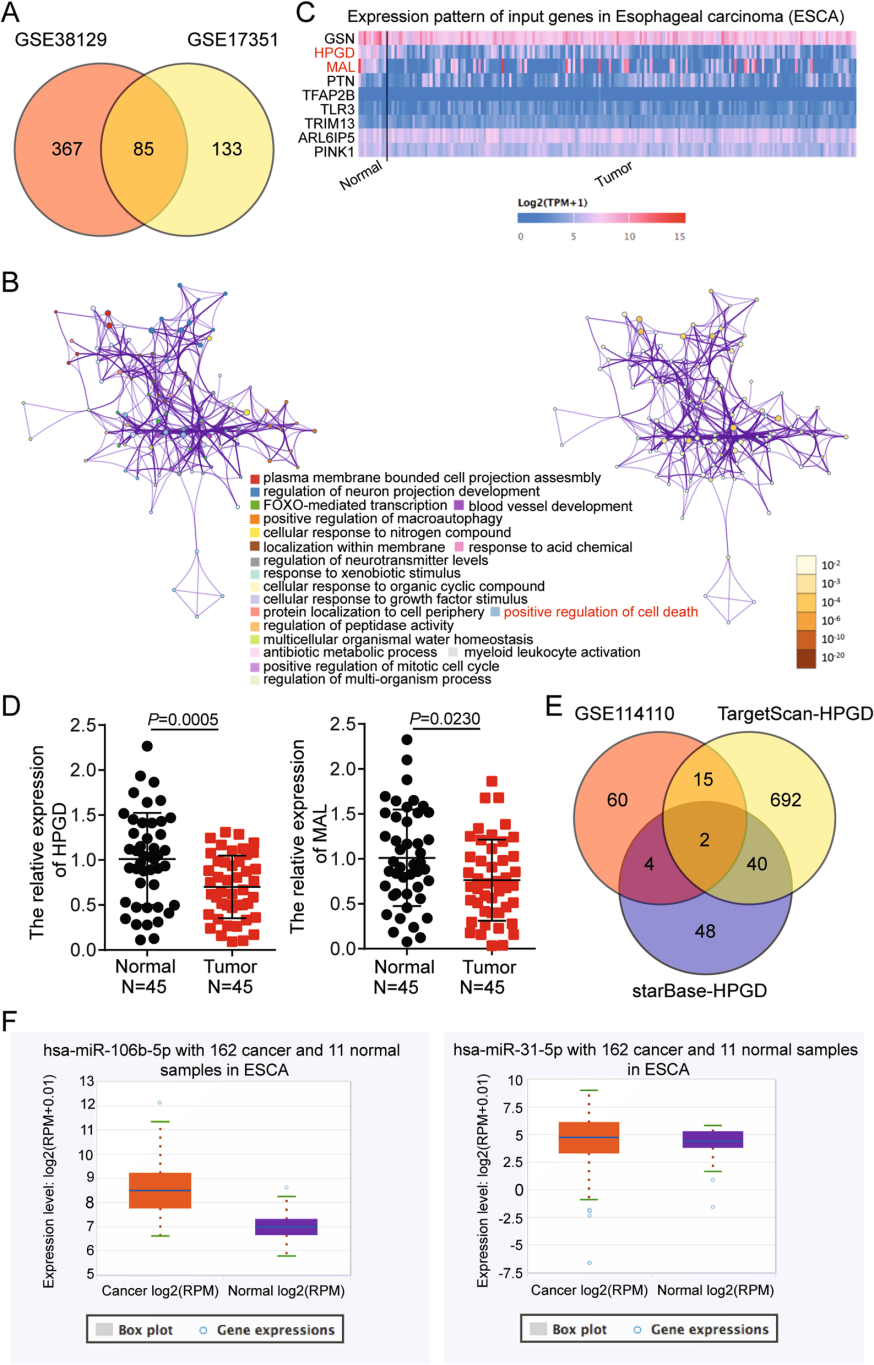
HPGD and miR-106b-5p were selected to be further investigate in ESCC by microarray analysis. **A** 85 DEGs were overlapped from GSE38129 and GSE17351 by Venny 2.1.0. GSE38129 and GSE17351 were the mRNA expression profiles. **B** The positive regulation of cell death containing 9 DEGs was screen out as the key progress by Metascape analysis. **C** The expression of HPGO and MAL in ESCA was significant reduced. **D** The expression of HPGO and MAL was detected in the ESCC tissue samples (*n* = 45) and normal tissues (*n* = 45) by qRT-PCR. **E** miR-106b-5p and miR-31-5p were overlapped from GSE114110, TargetScan, and starBase. GSE114110 was the miRNA expression microarray. TargetScan and starBase were used to predict the miRNAs targeting HPGD. **F** The expression of miR-106b-5p was significant down-regulated in ESCA by starBase analysis

HPGD and miR-106b-5p were screened using bioinformatics analysis, and a total of 452 and 218 DEGs were identified in the GSE38129 and GSE17351 databases, respectively. Using Venny 2.1.0 (Fig. 1A), 85 overlapping DEGs were identified in GSE38129 and GSE17351. Metascape was used to process the 85 DEGs, and the positive regulation of cell death containing nine DEGs was selected as the key biological process (Fig. 1B). HPGD and MAL RNA transcripts were significantly downregulated in ESCA (Fig. 1C). Analysis of the mRNA levels of HPGD and MAL in clinical tissues revealed that HPGD expression was lower compared to that of MAL in the ESCC tissues (Fig. 1D). Additionally, HPGD expression was found to be significantly associated with the histological differentiation, tumor-node-metastasis (TNM) stage, and karnofsky performance status (KPS), but was not associated with gender, age, and weight loss in 45 ESCC cases (Table 1). Therefore, HPGD was selected as the gene of interest and further investigated in ESCC. TargetScan and starBase, used to predict the miRNAs that target HPGD, identified 749 and 94 miRNAs, respectively. Based on Venny 2.1.0 analysis miR-106b-5p and miR-31-5p were shortlisted as molecules of interest due to their overlap in the GSE114110 miRNA microarray, TargetScan, and starBase (Fig. 1E). Compared to miR-31-5p, miR-106b-5p expression was significantly upregulated in ESCA samples, and therefore we focused on miR-106b-5p for further experiments (Fig. 1F).

### High expression of miR-106b-5p in ESCC

**Fig. 2:**
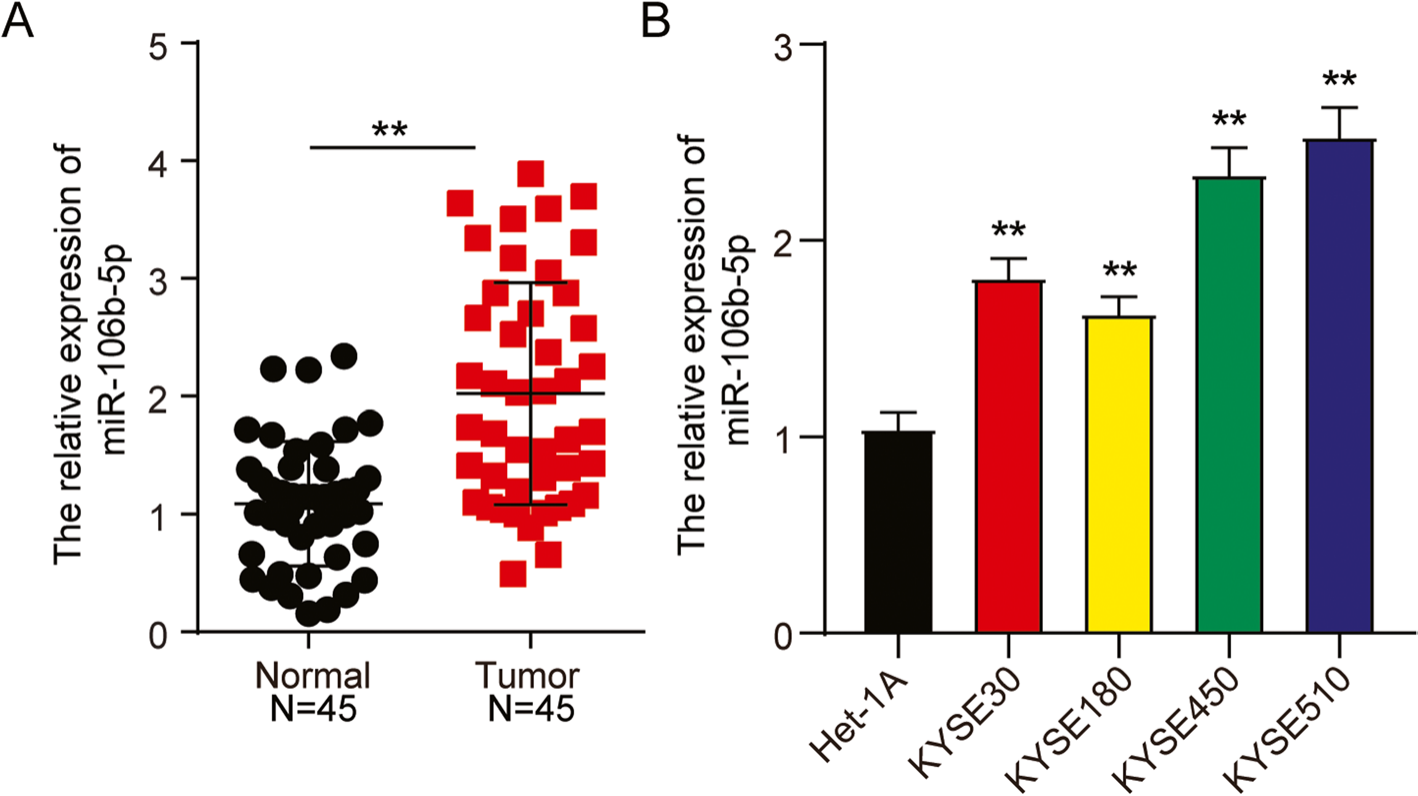
MiR-106b-5p was upregulated in ESCC tissues. **A** RT-qPCR detection of miR-106b-5p expression in ESCC tissues (*n* = 45) and normal tissues (*n* = 45). **, *P* < 0.001 compared to normal tissues. **B** Measurement of miR-106b-5p expression in ESCC cells lines (KYSE30, KYSE180, KYSE450, and KYSE510) and normal esophageal epithelial cells (Het-1A). Data are presented as means± SD of at least three independent tests per experiment. **, *P* < 0.001 compared to Het-1A cells

Aberrant upregulation of miR-106b-5p was detected by RT-qPCR analysis in ESCC tissues and cell lines. As shown in Fig. 2A, miR-106b-5p expression in the tumor tissues was nearly 2-fold higher than that in the corresponding normal tissues. The relationships between miR-106b-5p expression levels and the clinicopathological characteristics of individuals with ESCC are summarized in Table 1. We did not find a significant association between miR-106b-5p expression levels and patient gender, age, and weight loss in 45 ESCC cases. However, we found that the expression level of miR-106b-5p was positively correlated with histological differentiation, TNM stage, and KPS in ESCC patients. Examination of miR-106b-5p expression in ESCC cell lines revealed robust expression of miR-106b-5p in all ESCC cell lines compared to that in Het-1A cells (Fig. 2B). As KYSE450 and KYSE510 cells showed the highest miR-106b-5p expression levels, they were used in the subsequent experiments.

### Promotion of ESCC progression by miR-106b-5p

**Fig. 3:**
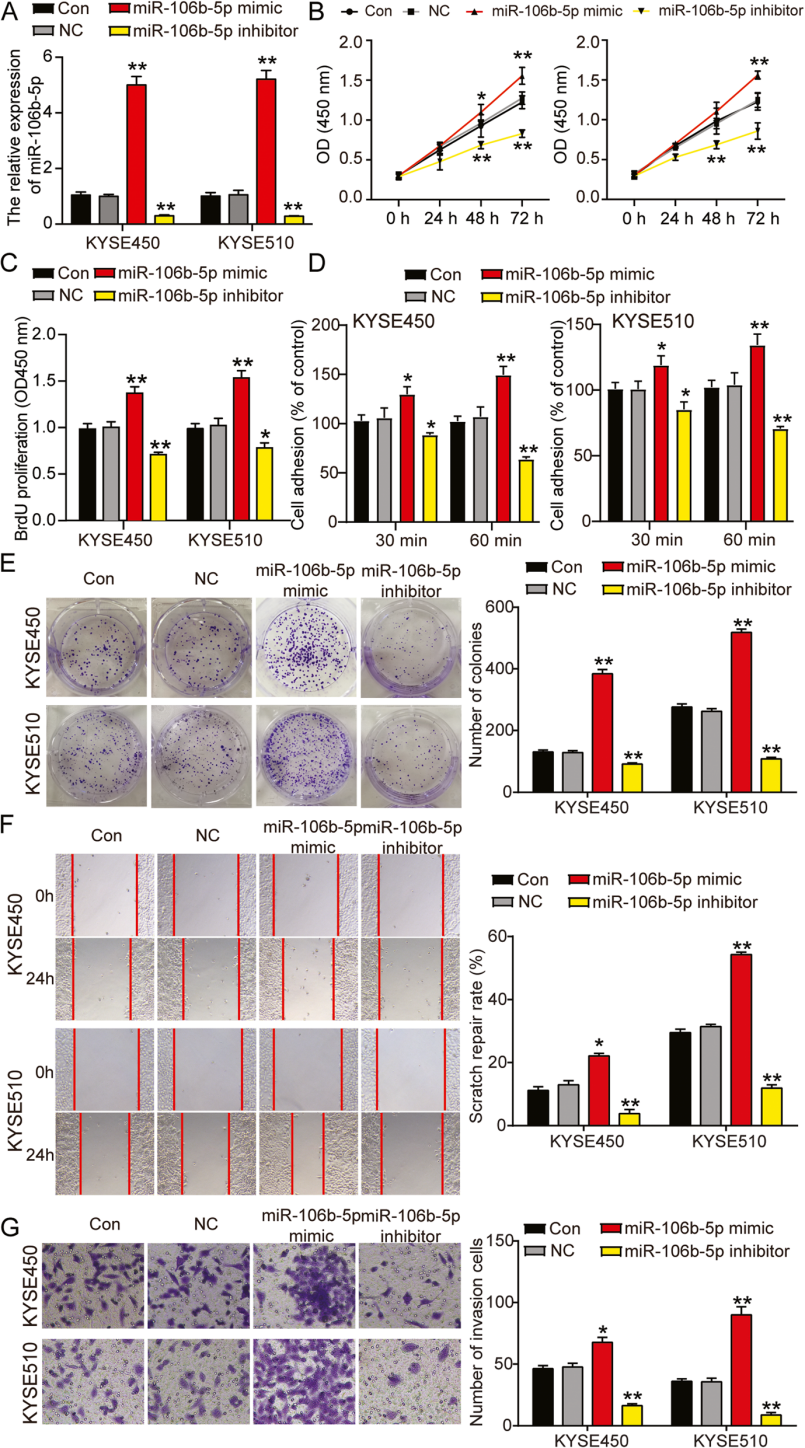
MiR-106b-5p promoted cell proliferation, adhesion, colony formation, migration and invasion in ESCC. **A** Measurement of miR-106b-5p expression in KYSE450 and KYSE510 cells transfected with NC, miR-106b-5p mimic, and miR-106b-5p inhibitor with RT-qPCR. **B** Cell viability was detected in KYSE450 and KYSE510 cells transfected with miR-106b-5p mimic, and miR-106b-5p inhibitor by CCK-8 assay. **C** Cell proliferation was detected in KYSE450 and KYSE510 cells transfected with, miR-106b-5p mimic, and miR-106b-5p inhibitor by BrdU assay. **D** Cell adhesion was detected in KYSE450 and KYSE510 cells transfected with NC, miR-106b-5p mimic, and miR-106b-5p inhibitor by cell adhesion assay kit. **E** Cell colony formation was detected in KYSE450 and KYSE510 cells transfected with NC, miR-106b-5p mimic, and miR-106b-5p inhibitor by colony formation assay. **F** Cell migration rate was detected in KYSE450 and KYSE510 cells transfected with NC, miR-106b-5p mimic, and miR-106b-5p inhibitor by cell wound healing assay. **G** Cell invasion was detected in KYSE450 and KYSE510 cells transfected with NC, miR-106b-5p mimic, and miR-106b-5p inhibitor by transwell assay. Data are presented as means± SD of at least three independent tests per experiment. *, *P* < 0.05; **, *P* < 0.001 compared to CON group. CON, blank control; NC, mimic-NC + inhibitor-NC

**Fig. 4:**
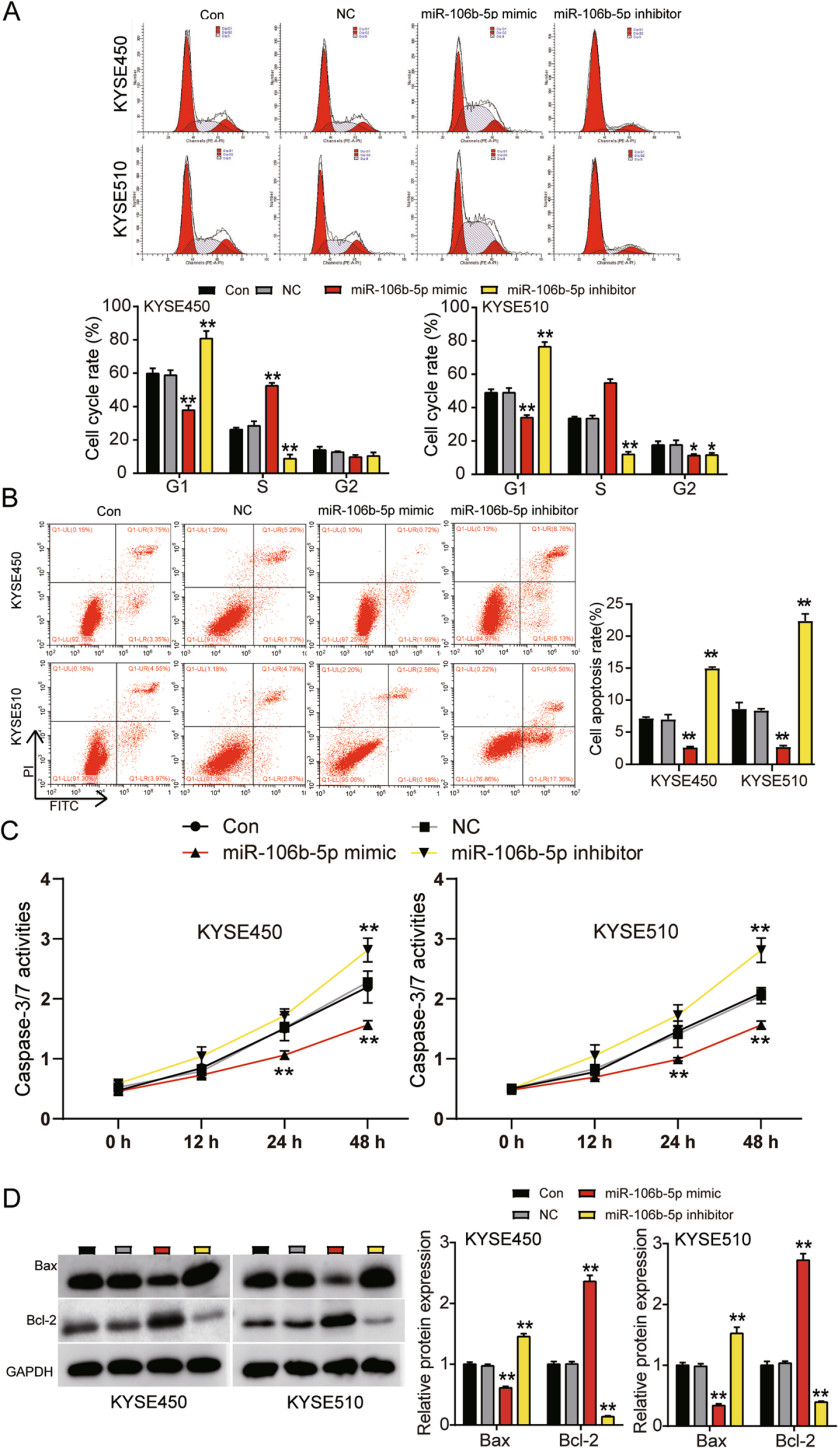
MiR-106b-5p promoted cell cycle progression and suppressed cell apoptosis in ESCC. **A** Cell cycle was detected in KYSE450 and KYSE510 cells transfected with NC, miR-106b-5p mimic, and miR-106b-5p inhibitor by flow cytometry assay. **B** Cell apoptosis rate was detected in KYSE450 and KYSE510 cells transfected with NC, miR-106b-5p mimic, and miR-106b-5p inhibitor by flow cytometry assay. **C** Cell apoptosis was determined in KYSE450 and KYSE510 cells transfected with NC, miR-106b-5p mimic, and miR-106b-5p inhibitor by caspase-3/7 activity assay kit. **D** The protein expression of Bax and Bcl-2 were determined in KYSE450 and KYSE510 cells transfected with NC, miR-106b-5p mimic, and miR-106b-5p inhibitor by western blot analysis. Data are presented as means± SD of at least three independent tests per experiment. *, *P* < 0.05; **, *P* < 0.001 compared to CON group. CON, blank control; NC, mimic-NC + inhibitor-NC

Synthetic target miRNA analogs were transfected into KYSE450 and KYSE510 cells to examine the effect of miR-106b-5p on ESCC cells. The transfection efficiency was determined by RT-qPCR (Fig. 3A and Supplemental Fig. 1). Further cellular functional assays demonstrated that the miR-106b-5p mimic increased cell viability and proliferation; whereas the miR-106b-5p inhibitor produced the opposite effect (Fig. 3B and C). Additionally, cell adhesion and colony formation experiments showed that upregulation of miR-106b-5p promoted adhesion and colony formation, and inhibition of miR-106b-5p reduced adhesion and colony formation (Fig. 3D and E). Likewise, an increase in cell migration and invasion was observed in wound healing and Transwell assays following transfection with the miR-106b-5p mimic. In contrast, the miR-106b-5p inhibitor impaired cell migration and invasion (Fig. 3F and G). Flow cytometry was performed to analyze the cell cycle and apoptosis rate. As shown in Fig. 4A and B, overexpression of miR-106b-5p decreased the proportion of cells in G1 phase and the apoptosis rate, and increased the proportion of cells in S-phase. In constrast, knockdown of miR-106b-5p showed an opposite effect on cell cycle and apoptosis. Similarly, analysis of the caspase-3/7 activity revealed that treatment with the miR-106b-5p inhibitor resulted in increased caspase-3/7 activity at 24 h, whereas the activity was reduced in the miR-106b-5p mimic group (Fig. 4C). Treatment with the miR-106b-5p mimic also reduced Bax protein levels and elevated Bcl-2 protein levels compared to that in the untransfected cells, whereas the miR-106b-5p inhibitor showed the opposite effect in the two ESCC cell lines (Fig. 4D).

### Inhibition of miR-106b-5p reduced the growth of ESCC xenografts and pulmonary metastasis

**Fig. 5:**
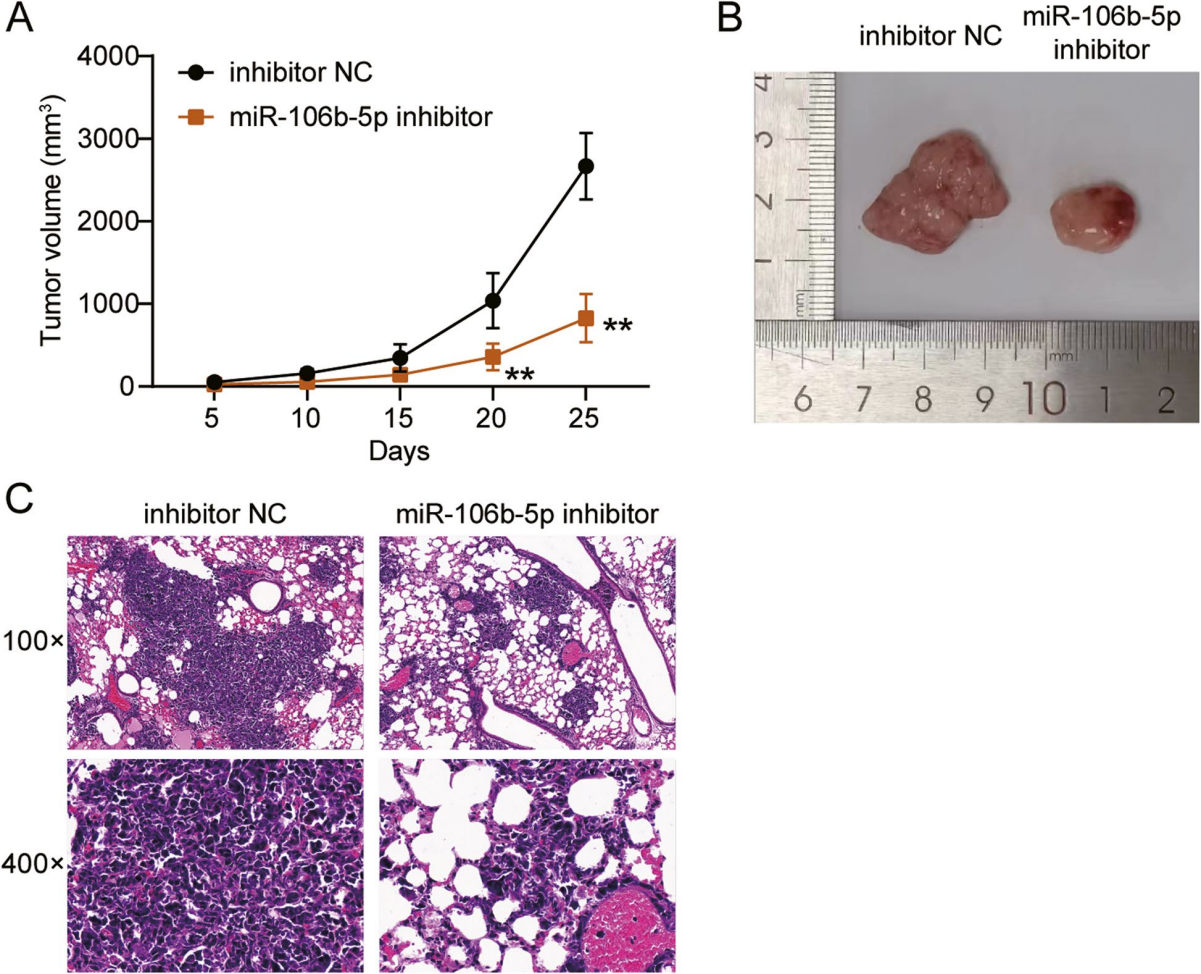
MiR-106b-5p promoted s xenograft and pulmonary metastasis in ESCC. **A** Tumor growth curves measured after the inoculation of nude mice injected with KYSE510 cells transfected with inhibitor-NC, and miR-106b-5p inhibitor. **B** Photographs of tumors in nude mice injected with KYSE510 cells transfected with inhibitor-NC, and miR-106b-5p inhibitor. **C** Representative photographs of H&E stained spontaneous lung metastases in nude mice injected with KYSE510 cells transfected with inhibitor-NC, and miR-106b-5p inhibitor

To analyze the effect of miR-106b-5p on tumor growth and pulmonary metastasis of ESCC cells, KYSE510 cells that with miR-106b-5p inhibition were injected into nude mice. The results showed that tumor volume increased with time in the control group, whereas inhibition of miR-106b-5p reduced the tumor volume (Fig. 5A). This was further confirmed by visual observation of the tumor sizes following excision (Fig. 5B). Moreover, H&E staining was performed on the lung tissues following resection from the mice to observe the degree of metastasis, which indicated that reduced expression of miR-106b-5p inhibited tumor metastasis compared with to that in the control group (Fig. 5C).

### Effect of miR-106b-5p on HPGD expression

**Fig. 6:**
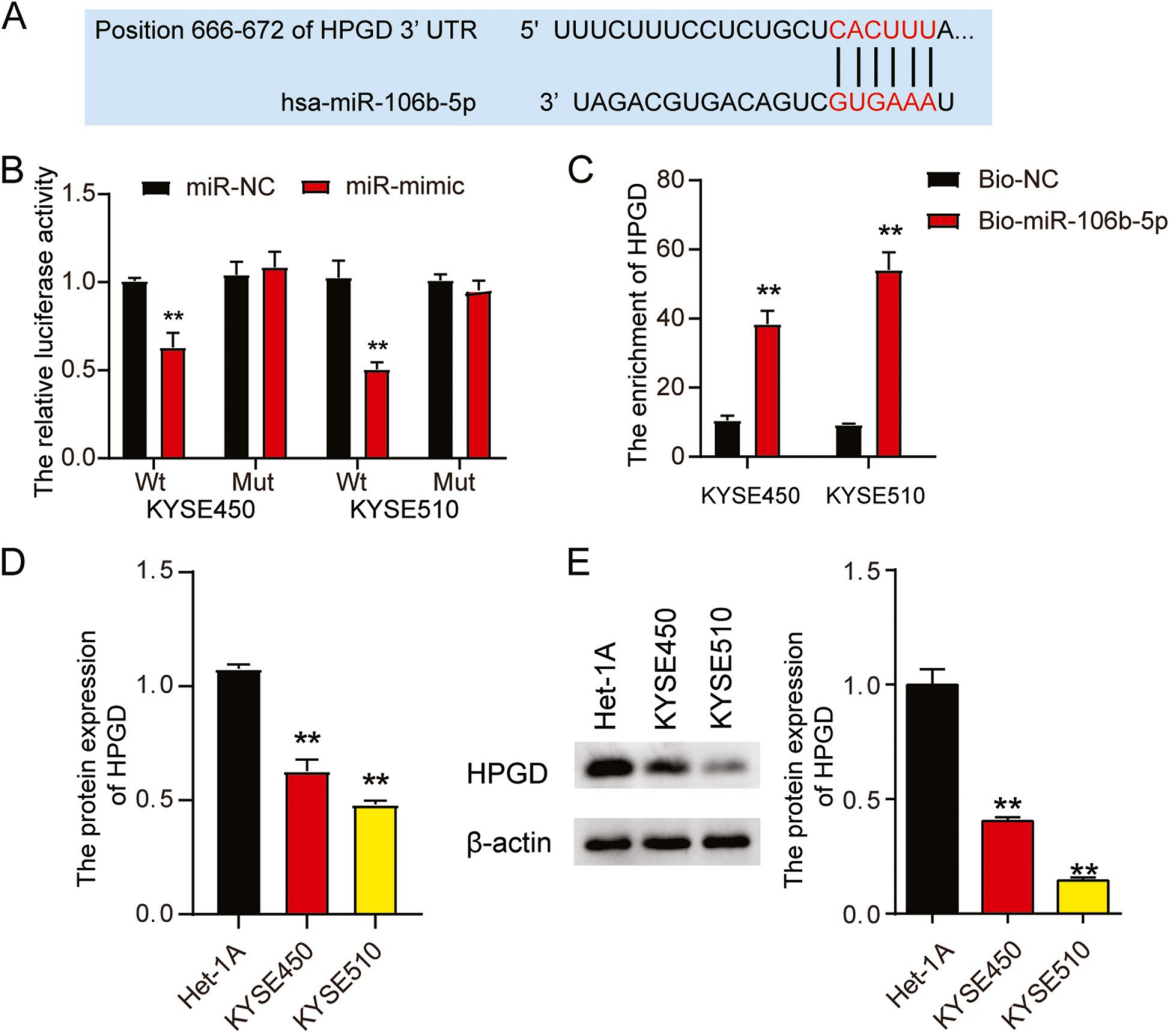
HPGD was a direct target ofmiR-106b-5p. **A** Bioinformatics analysis showed the predicted binding sequence of HPGD 3’-UTR. **B** Dual luciferase assay was performed in cells co-transfected with plasmids HPGD-Wt or HPGD-Mut and miR-NC or miR-106b-5p mimic in KYSE450 and KYSE510 cells. **, *P* < 0.001 compared to co-transfection of HPGD-Wt and miR-NC group. **C** RNA pull down assay was used to detect the association between HPGD and miR-106b-5p in KYSE450 and KYSE510 cells. **, *P* < 0.001 compared to Bio-NC group. **D** RT-qPCR detection of expression of HPGD in Het-1A, KYSE450 and KYSE510 cells. **, *P* < 0.001 compared to Het-1A cells. **E** Western blot detection of protein expression of HPGD in Het-1A, KYSE450 and KYSE510 cells. Data are presented as means ± SD of at least three independent tests per experiment. *, *P* < 0.05; **, *P* < 0.001 compared to Het-1A cells. Wt, wild-type; Mut, mutant; NC, negative control

The potential binding sequence of miR-106b-5p in the 3’UTR of HPGD was predicted using TargetScan Human 7.2 (Fig. 6A). Luciferase reporter assays revealed that transfection with the miR-106b-5p-mimic reduced the luciferase signals in both the ESCC cell lines with wild-type HPGD 3’-UTR plasmid; however, it did not affect the luciferase activity in the cells transfected with the mutant HPGD3’-UTR plasmid (Fig. 6B), indicating that miR-106b-5p directly targets HPGD. The physical interaction between miR-106b-5p and HPGD was also demonstrated using an RNA pull-down assay (Fig. 6C). Furthermore, HPGD expression was reduced by 30–50% in both the ESCC cells compared to that in Het-1A cells, as evidenced by RT-qPCR and western blotting (Fig. 6D and E). Taken together, these results indicated that HPGD is potential a target gene of miR-106b-5p.

### Promotion of ESCC progression by miR-106b-5p/HPGD axis

**Fig. 7:**
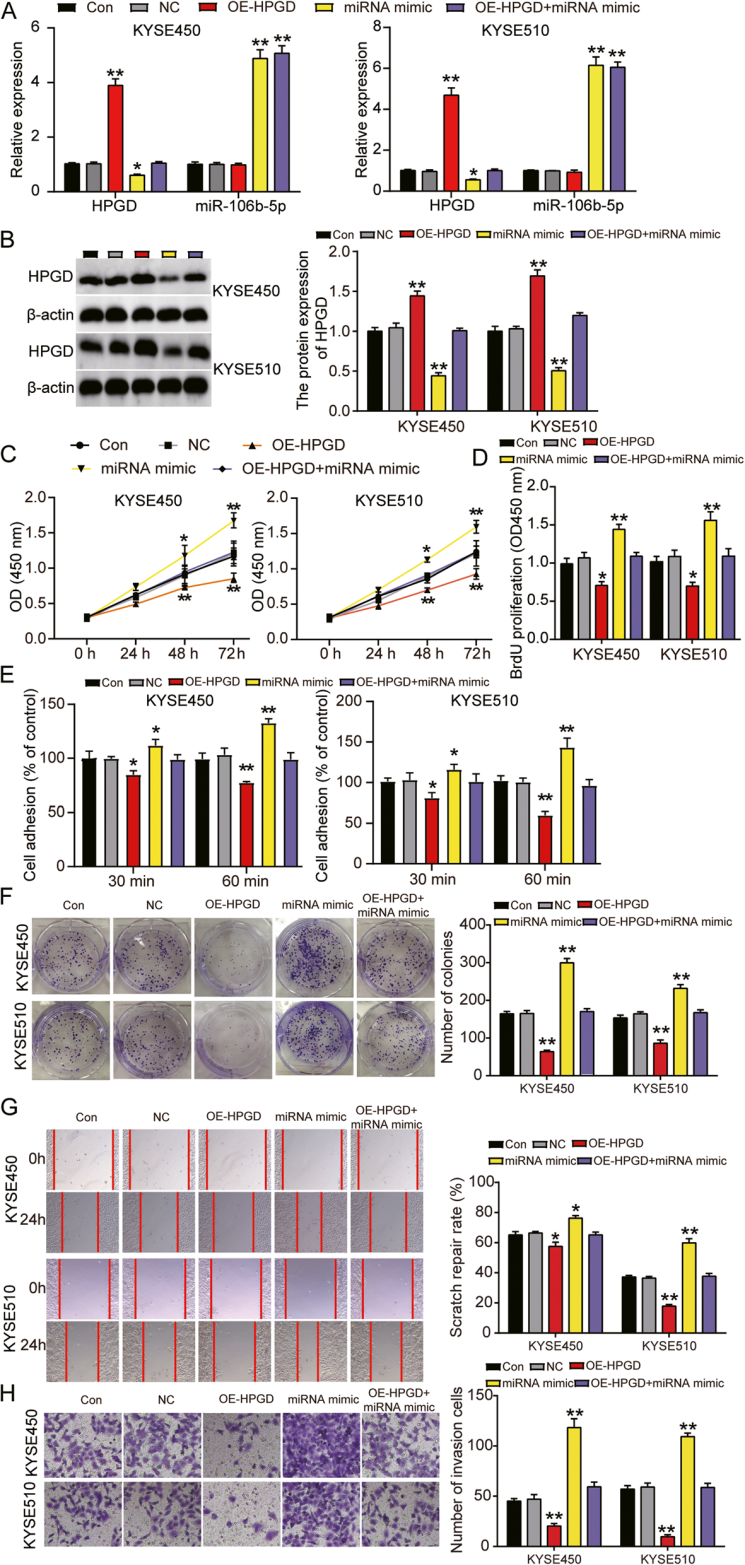
MiR-106b-5p targeting to HPGD promoted proliferation, adhesion, colony formation, migration and invasion of ESCC cells. **A** Measurement of HPGD and miR-106b-5p gene expression in KYSE450 and KYSE510 cells transfected with NC, OE-HPGD, miR-106b-5p mimic, and OE-HPGD+miR-106b-5p mimic by RT-qPCR. **B** Measurement of HPGD protein expression in KYSE450 and KYSE510 cells transfected with NC, OE-HPGD, miR-106b-5p mimic, and OE-HPGD+miR-106b-5p mimic by western blot. **C** Cell viability was detected in KYSE450 and KYSE510 cells transfected with NC, OE-HPGD, miR-106b-5p mimic, and OE-HPGD+miR-106b-5p mimic by CCK-8 assay. **D** Cell proliferation was detected in KYSE450 and KYSE510 cells transfected with NC, OE-HPGD, miR-106b-5p mimic, and OE-HPGD+miR-106b-5p mimic by BrdU assay. **E** Cell adhesion was detected in KYSE450 and KYSE510 cells transfected with NC, OE-HPGD, miR-106b-5p mimic, and OE-HPGD+miR-106b-5p mimic by cell adhesion assay kit. **F** Cell colony formation was detected in KYSE450 and KYSE510 cells transfected with NC, OE-HPGD, miR-106b-5p mimic, and OE-HPGD+miR-106b-5p mimic by colony formation assay. **G** Cell migration rate was detected in KYSE450 and KYSE510 cells transfected with NC, OE-HPGD, miR-106b-5p mimic, and OE-HPGD+miR-106b-5p mimic by cell wound healing assay. **H** Cell invasion was detected in KYSE450 and KYSE510 cells transfected with NC, OE-HPGD, miR-106b-5p mimic, and OE-HPGD+miR-106b-5p mimic by transwell assay. Data are presented as means± SD of at least three independent tests per experiment. *, *P* < 0.05; **, *P* < 0.001 compared to CON group. CON, blank control; NC, empty vectors+mimic-NC; OE-HPGD, overexpression-HPGD; OE-HPGD+miR-106b-5p mimic, overexpression-HPGD+ miR-106b-5p mimic

**Fig. 8:**
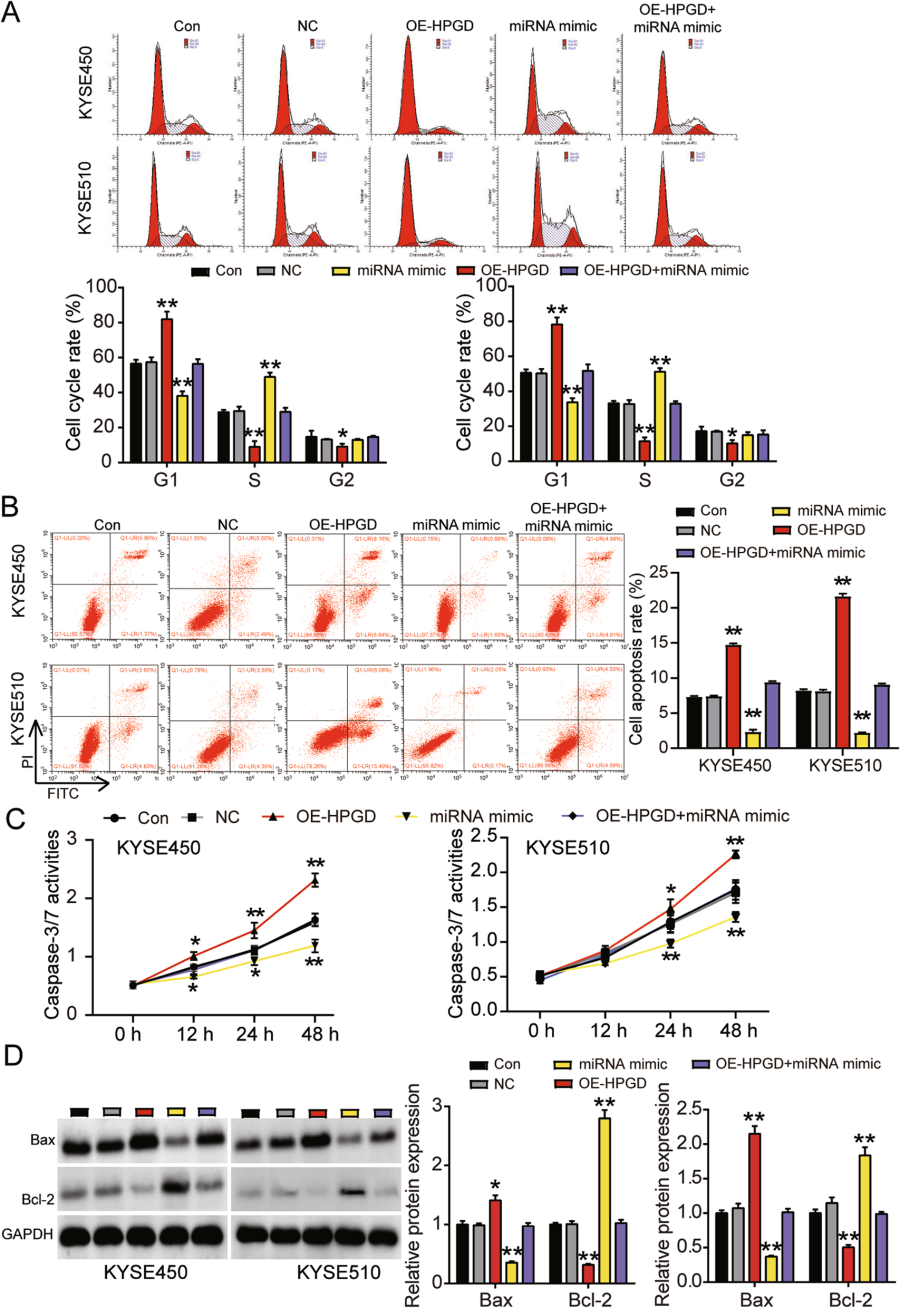
MiR-106b-5p targeting to HPGD promoted cell cycle progression and suppressed cell apoptosis of ESCC cells. **A** Cell cycle was detected in KYSE450 and KYSE510 cells transfected with NC, OE-HPGD, miR-106b-5p mimic, and OE-HPGD+miR-106b-5p mimic by flow cytometry assay. **B** Cell apoptosis rate was detected in KYSE450 and KYSE510 cells transfected with NC, OE-HPGD, miR-106b-5p mimic, and OE-HPGD+miR-106b-5p mimic by flow cytometry assay. **C** Cell apoptosis was determined in KYSE450 and KYSE510 cells transfected with NC, OE-HPGD, miR-106b-5p mimic, and OE-HPGD+miR-106b-5p mimic by caspase-3/7 activity assay kit. **D** The protein expression of Bax and Bcl-2 were determined in KYSE450 and KYSE510 cells transfected with NC, OE-HPGD, miR-106b-5p mimic, and OE-HPGD+miR-106b-5p mimic by western blot analysis. Data are presented as means± SD of at least three independent tests per experiment. *, *P* < 0.05; **, *P* < 0.001 compared to CON group. CON, blank control; NC, empty vectors+mimic-NC; OE-HPGD, overexpression-HPGD; OE-HPGD+miR-106b-5p mimic, overexpression-HPGD+ miR-106b-5p mimic

KYSE450 and KYSE510 cells were transfected with HPGD overexpression plasmid (OE-HPGD) and miR-106b-5p-mimic to further explore the underlying mechanism of the miR-106b-5p/HPGD axis. Prior to the functional assays, the transfection efficiency of OE-HPGD was validated by RT-qPCR and western blotting (Supplemental Fig. 2A and B). RT-qPCR confirmed that HPGD expression increased by 4-fold in the cells transfected with OE-HPGD, and decreased by 50% following co-transfection with miR-106b-5p mimic. However, this increased HPGD protein expression was abrogated by the miR-106b-5p-mimic. The miR-106b-5p mimic increased miR-106b-5p expression by 5-fold; however, OE-HPGD had no effect on miR-106b-5p expression (Fig. 7A). Western blot analysis of HPGD protein levels showed similar results as that observed in RT-qPCR (Fig. 7B). The viability and proliferation of cells transfected with OE-HPGD were significantly inhibited, whereas that of cells transfected with miR-106b-5p mimic were markedly enhanced. Furthermore, the miR-106b-5p-mimic relieved the inhibitory effect of OE-HPGD on cell proliferation (Fig. 7C and D). Transfection with miR-106b-5p mimic enhanced adhesion and colony formation ability, whereas OE-HPGD decreased the adhesion and colony formation ability of the cells. Additionally, the miR-106b-5p-mimic reversed the OE-HPGD-induced changes in cell adhesion and colony formation (Fig. 7E and F). In addition, wound healing and Transwell assays showed that overexpression of HPGD inhibited the migration and invasion of the cancer cells, and reversed the ability of miR-106b-5p mimic to promote migration and invasion of the cancer cells (Fig. 7G and H). Flow cytometric analysis showed that upregulation of HPGD partially eliminated the effect of miR-106b-5p overexpression on the cell cycle and apoptosis in KYSE450 and KYSE510 cells, and decreased the proportion of cells in G1 phase and apoptosis, and increased the proportion of cells in G2 phase (Fig. 8A and B). Moreover, results of the caspase-3/7 activity and western blot assays revealed that miR-106b-5p overexpression markedly reduced caspase-3/7 activity and Bax protein level and enhanced Bcl-2 protein level. In contrast, HPGD overexpression increased caspase-3/7 activity and Bax protein level and decreased Bcl-2 protein level. Furthermore, we found that the miR-106b-5p-mimic inhibited the elevated caspase-3/7 activity and Bax protein level, and decreased Bcl-2 protein level induced by OE-HPGD (Fig. 8C and D). Overall, these data indicated that miR-106b-5p enhanced ESCC progression by targeting HPGD.

## Discussion

MiR-106b-5p has been implicated in several types of cancers [22, 29]. In this study, we provide data to support that miR-106b-5p has a tumor-promoting effect and mediates tumor progression. Using bioinformatics analysis, we first identified miR-31-5p and miR-106b-5p as two potential miRNAs that target HPGD and participate in ESCC. Because of its higher differential expression in ESCC tissues compared to miR-31-5p, we further investigated the function of miR-106b-5p. Our findings showed an increase in miR-106b-5p expression and a decrease in HPGD expression in ESCC samples. In addition, miR-106b-5p inhibited the expression of HPGD at both mRNA and protein levels. In addition, miR-106b-5p promoted proliferation, colony formation, adhesion, migration, and invasion, and induced cell cycle, but inhibited apoptosis of ESCC cells. Silencing of miR-106-5p inhibited tumor growth and lung metastasis in vivo.

A previous study documented the ability of miR-106b-5p to inhibit the invasion and metastasis of colorectal cancer (CRC) cells [17]. In another study, low expression of miR-106b-5p correlated with poor survival of patients with CRC [21]. Only a single study reported that miR-106b-5p promotes cell migration and invasion via enhancement of EMT by targeting SMAD family member 7 (Smad7) [25]. As cell viability, proliferation, adhesion, and apoptosis were the key characteristics of cancer cells [22, 30, 31], we evaluated these cellular functions through a series of assays. Consistently, upregulation of miR-106b-5p was observed in ESCC tissues and cells that promoted the progression of ESCC by enhancing the viability, proliferation, colony formation, adhesion, migration, and invasive ability of the cells, and induced cell cycle progression and suppressed apoptosis of ESCC cells. We also showed that silencing of miR-106-5p inhibited tumor growth and lung metastasis in vivo.

Several studies have demonstrated that HPGD functions as a tumor suppressor gene in various cancers [8–10]. The downregulation of HPGD caused by activation of interleukin-1β led to a poor prognosis in pancreatic cancer patients, and reduction of HPGD expression was associated with tumorigenesis [8]. In the context of breast cancer, HPGD acts as a tumor suppressor, and the upregulation of HPGD was found to reduce tumorigenesis in athymic mice [9]. Another study showed that HPGD expression and activity was decreased in CRC tissues [10]. However, a previous studies reported a decrease in HPGD expression in ESCC tissues [11], and in an isolated human metastasizing esophageal cancer cell line [32], suggesting that HPGD may contribute to ESCC development. Based on previous studies and our bioinformatics analysis, we suspected that HPGD was a potential tumor suppressor gene in ESCC and that miR-106b-5p may bind to the 3’UTR of HPGD to regulate ESCC progression. Cytological assays revealed that miR-106b-5p contributed to the progression of ESCC by enhancing cell proliferation, colony formation, adhesion, migration, and invasion, and induced cell cycle progression, while inhibiting cell apoptosis. In addition, a previous study demonstrated that HPGD suppresses colon cancer aggressiveness through the STAT3 and AKT pathways [33]. Therefore, future studies should investigate this pathway to confirm this interaction in ESCC.

Several studies have shown that each miRNA targets multiple mRNAs [34]. Furthermore, miR-106b-5p has been reported to target different mRNAs in a variety of cancers. For example, Gao et al. [35] found that miR-106b-5p regulates the growth of clear cell renal cell carcinoma by targeting PDCD. Another study revealed that miR-106b-5p regulates the migration and invasion of CRC cells by targeting FAT4 [36]. Therefore, in future research, we need to further study other mRNA targets that are regulated by miR-106b-5p in ESCC, to better understand the miRNA-mRNA regulatory networks in ESCC.

Immortalized cell lines and primary cells are indispensable tools in the study of cancer pathogenesis and have been shown to share similar pathophysiological changes [37]. However, some studies have found that there are some differences between cell lines and primary cells in their protein properties, morphology, and metabolic activity [38]. Therefore, it is necessary to isolate and culture primary cancer cells from tumor tissues of ESCC patients to further understand the effects of miR-106b-5p/HPGD in ESCC. In addition, the epidemiology and pathogenesis of ESCC varies worldwide [39]. Our study only investigated the association between the expression of miR-106b-5p and HPGD, and the characteristics of patients from China. To identify biomarkers of ESCC, the association between the expression of miR-106b-5p and HPGD and characteristics needs to be analyzed in patients from different parts of the world.

## Conclusion

In summary, our data suggest that miR-106b-5p and HPGD affect ESCC progression. More specifically, our findings indicated that miR-106b-5p accelerates proliferation, colony formation, adhesion, migration, invasion, and induces cell cycle progression, but represses apoptosis in vitro by targeting HPGD. In vivo, silencing of miR-106b-5p inhibits tumor growth and lung metastasis. Thus, miR-106b-5p and HPGD represent promising targets for the diagnosis and treatment of ESCC.

## Supplementary Information

**Additional file 1: Supplemental Figure 1.** The measurement of miR-106b-5p expression in KYSE450 and KYSE510 cells transfected with mimic-NC and/or inhibitor-NC, miR-106b-5p mimic or miR-106b-5p mimic by RT-qPCR. Data are presented as means± SD of at least three independent tests per experiment. *, *P* < 0.05; **, *P* < 0.001 compared to CON group. CON, blank control; mimic-NC, miRNA mimic corresponding negative control; inhibitor-NC, miRNA inhibitor corresponding negative control; co-NC, mimic-NC + inhibitor-NC; miRNA mimic, miR-106b-5p mimic; miRNA inhibitor, miR-106b-5p inhibitor. **Supplemental Figure 2.** (A) Measurement of HPGD mRNA expression in KYSE450 and KYSE510 cells transfected with empty vector and/or mimic-NC or OE-HPGD by RT-qPCR. (B) Measurement of HPGD protein expression in KYSE450 and KYSE510 cells transfected with empty vector and/or mimic-NC or OE-HPGD by western blot. Data are presented as means± SD of at least three independent tests per experiment. *, *P* < 0.05; **, *P* < 0.001 compared to CON group. CON, blank control; empty vecor, pcDNA3.1 empty vector; mimic-NC, miRNA mimic corresponding negative control; co-NC, empty vector+mimic-NC; OE-HPGD, overexpression-HPGD.

**Additional file 2: Supplemental Table 1.** Sequences for cell transfection.

## Data Availability

Not applicable.

## Abbreviations

adj. P: Adjusted *P*-value
Bio-miR-106b-5p: Biotin-labeled miR-106b-5p
Bio-NC: Biotin-labeled negative control
DEGs: Differentially expressed genes
EC: Esophageal cancer
EMT: Epithelial-mesenchymal transition
ESCA: Esophageal carcinoma
ESCC: Esophageal squamous cell carcinoma
HPGD: Hydroxyprostaglandin dehydrogenase
logFC: Log fold change
miRNAs: microRNAs
OE-HPGD: Overexpression plasmids
PVDF: Polyvinylidene difluoride
SD: Standard deviation
Smad7: SMAD Family Member 7
TCGA: The Cancer Genome Atlas
