# Supplementary material for: MiR-106b-5p regulates esophageal squamous cell carcinoma progression by binding to HPGD
Source: BMC Cancer. 2022 Mar 22;22:308. doi: 10.1186/s12885-022-09404-8 (PMC8941792; doi:10.1186/s12885-022-09404-8)

**Supplemental Figure 1.** The measurement of miR-106b-5p expression in KYSE450 and KYSE510 cells transfected with mimic-NC and/or inhibitor-NC, miR-106b-5p mimic or miR-106b-5p mimic by RT-qPCR. Data are presented as means± SD of at least three independent tests per experiment. *, P< 0.05; **, P< 0.001 compared to CON group. CON, blank control; mimic-NC, miRNA mimic corresponding negative control; inhibitor-NC, miRNA inhibitor corresponding negative control; co-NC, mimic-NC+inhibitor-NC; miRNA mimic, miR-106b-5p mimic; miRNA inhibitor, miR-106b-5p inhibitor.


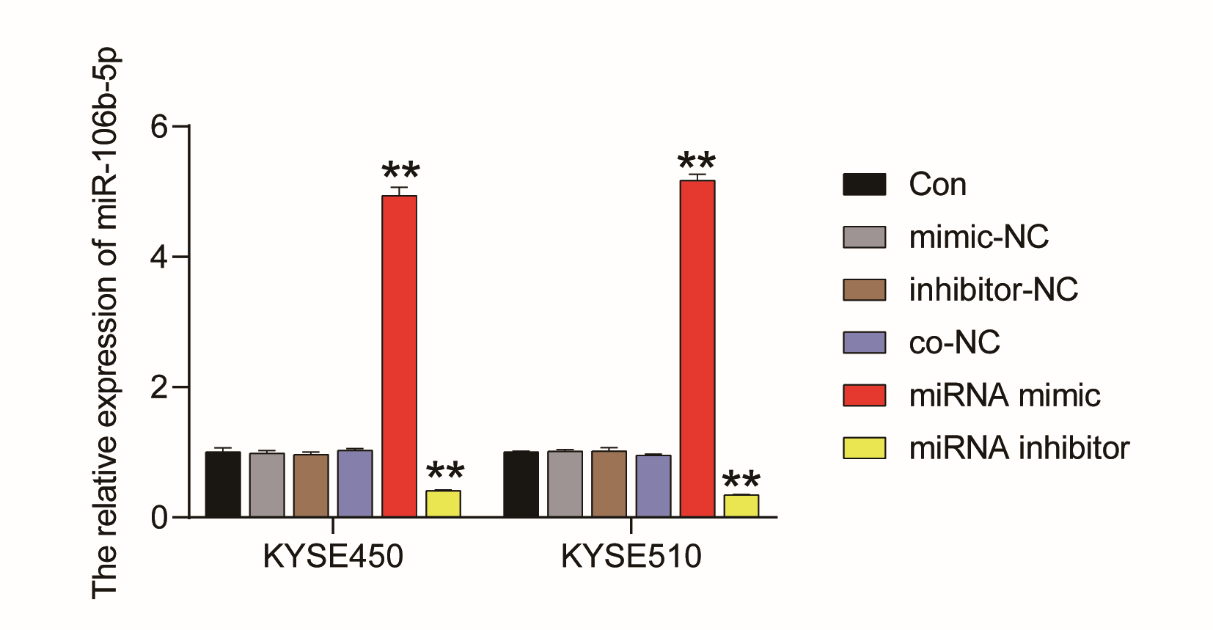


**Supplemental Figure 2.** (A) Measurement of HPGD mRNA expression in KYSE450 and KYSE510 cells transfected with empty vector and/or mimic-NC or OE-HPGD by RT-qPCR. (B) Measurement of HPGD protein expression in KYSE450 and KYSE510 cells transfected with empty vector and/or mimic-NC or OE-HPGD by western blot. Data are presented as means± SD of at least three independent tests per experiment. *, P< 0.05; **, P< 0.001 compared to CON group. CON, blank control; empty vecor, pcDNA3.1 empty vector; mimic-NC, miRNA mimic corresponding negative control; co-NC, empty vector+mimic-NC; OE-HPGD, overexpression-HPGD.


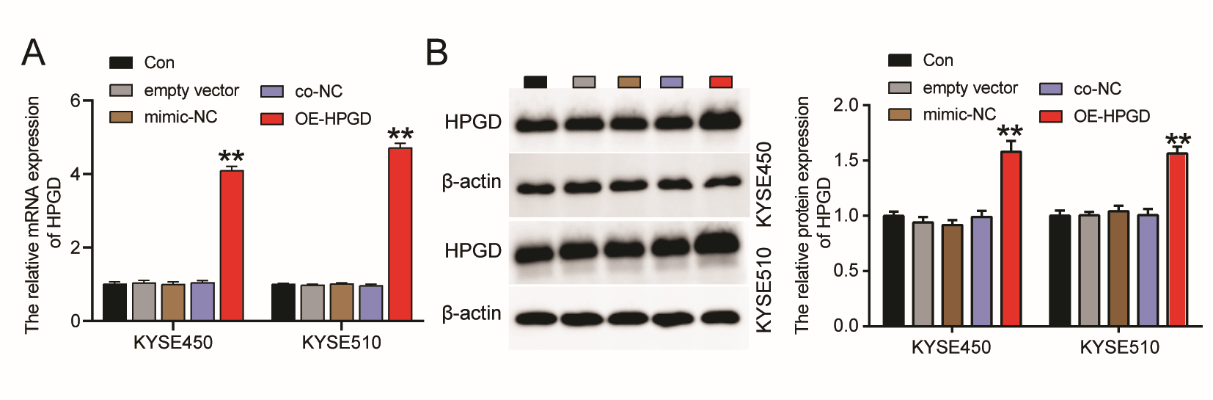

Supplement: Supplementary file 1 — Additional file 1: Supplemental Figure 1. The measurement of miR-106b-5p expression in KYSE450 and KYSE510 cells transfected with mimic-NC and/or inhibitor-NC, miR-106b-5p mimic or miR-106b-5p mimic by RT-qPCR. Data are presented as means± SD of at least three independent tests per experiment. *, P < 0.05; **, P < 0.001 compared to CON group. CON, blank control; mimic-NC, miRNA mimic corresponding negative control; inhibitor-NC, miRNA inhibitor corresponding negative control; co-NC, mimic-NC + inhibitor-NC; miRNA mimic, miR-106b-5p mimic; miRNA inhibitor, miR-106b-5p inhibitor. Supplemental Figure 2. (A) Measurement of HPGD mRNA expression in KYSE450 and KYSE510 cells transfected with empty vector and/or mimic-NC or OE-HPGD by RT-qPCR. (B) Measurement of HPGD protein expression in KYSE450 and KYSE510 cells transfected with empty vector and/or mimic-NC or OE-HPGD by western blot. Data are presented as means± SD of at least three independent tests per experiment. *, P < 0.05; **, P < 0.001 compared to CON group. CON, blank control; empty vecor, pcDNA3.1 empty vector; mimic-NC, miRNA mimic corresponding negative control; co-NC, empty vector+mimic-NC; OE-HPGD, overexpression-HPGD. [file 12885_2022_9404_MOESM1_ESM.docx]
