# Supplementary material for: MiR-106b-5p regulates esophageal squamous cell carcinoma progression by binding to HPGD
Source: BMC Cancer. 2022 Mar 22;22:308. doi: 10.1186/s12885-022-09404-8 (PMC8941792; doi:10.1186/s12885-022-09404-8)
Supplement: Supplementary file 2 — Additional file 2: Supplemental Table 1. Sequences for cell transfection. [file 12885_2022_9404_MOESM2_ESM.docx]

Supplemental Table 1 Sequences for cell transfection

| Name | Sequences |
| --- | --- |
| miR-106b-5p mimic | Sense: 5’-UAAAGUGCUGACAGUGCAGAU-3’ |
| miR-106b-5p inhibitor | Sense: 5’-AUCUGCACUGUCAGCACUUUA-3’ |
| miR-mimic NC | Forward:5’-UUCUCCGAACGUGUCACGUTT-3’ |
| miR-inhibitor NC | Forward:5’-CAGUACUUUUGUGUAGUACAA-3’ |
